# 4-({(*E*)-[2-(But-3-en-1-yl)-1-(prop-2-en-1-yl)-4-sulfanyl-1*H*-imidazol-5-yl]methyl­idene}amino)-3-phenyl-1*H*-1,2,4-triazole-5(4*H*)-thione

**DOI:** 10.1107/S1600536811039833

**Published:** 2011-10-05

**Authors:** Sampath Natarajan, Rita Mathews

**Affiliations:** a Department of Advanced Technology Fusion, Konkuk University, 1 Hwayang-dong, Gwangjin-gu, Seoul 143 701, Republic of Korea

## Abstract

In the title compound, C_19_H_20_N_6_S_2_, the dihedral angle between the phenyl and triazole rings is 24.1 (2)° while the dihedral angles between the imidazole ring and the triazole and phenyl rings are 39.9 (2) and 55.3 (2)°, respectively. The crystal structure is stabilized by inter­molecular N—H⋯N hydrogen bonds which form chains along [10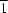].

## Related literature

For biological applications of Schiff base compounds, see: Liang (2003 ▶); Bacci *et al.* (2005 ▶). For the biological activity of triazoles and their derivatives, see: Amir *et al.* (2008 ▶); Sztanke *et al.* (2008 ▶); Padmavathi *et al.* (2008 ▶); Thenmozhi *et al.* (2010 ▶). Pharmacological compounds having triazole moieties appear to be very effective aromatese inhibitors for the prevention of breast cancer, see: Ünver *et al.* (2010 ▶).
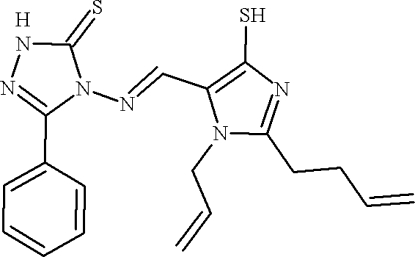


## Experimental

### 

#### Crystal data

C_19_H_20_N_6_S_2_
*M* *_r_* = 396.53
Monoclinic, 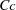

*a* = 13.384 (3) Å
*b* = 13.892 (3) Å
*c* = 11.349 (2) Å
β = 101.953 (3)°
*V* = 2064.5 (7) Å^3^
*Z* = 4
Mo *K*α radiation
μ = 0.27 mm^−1^
*T* = 293 K
0.28 × 0.25 × 0.23 mm

#### Data collection

Bruker SMART APEX CCD area-detector diffractometer
7754 measured reflections
3788 independent reflections
3391 reflections with *I* > 2σ(*I*)
*R*_int_ = 0.017

#### Refinement

*R*[*F* ^2^ > 2σ(*F* ^2^)] = 0.053
*wR*(*F* ^2^) = 0.139
*S* = 1.07
3788 reflections
244 parameters
2 restraints
H-atom parameters constrained
Δρ_max_ = 0.33 e Å^−3^
Δρ_min_ = −0.21 e Å^−3^

### 

Data collection: *APEX2* (Bruker, 2004 ▶); cell refinement: *SAINT* (Bruker, 2004 ▶); data reduction: *SAINT*; program(s) used to solve structure: *SHELXS97* (Sheldrick, 2008 ▶); program(s) used to refine structure: *SHELXL97* (Sheldrick, 2008 ▶); molecular graphics: *ORTEP-3* (Farrugia, 1997 ▶); software used to prepare material for publication: *PLATON* (Spek, 2009 ▶).

## Supplementary Material

Crystal structure: contains datablock(s) I, global. DOI: 10.1107/S1600536811039833/kj2187sup1.cif

Structure factors: contains datablock(s) I. DOI: 10.1107/S1600536811039833/kj2187Isup2.hkl

Supplementary material file. DOI: 10.1107/S1600536811039833/kj2187Isup3.cml

Additional supplementary materials:  crystallographic information; 3D view; checkCIF report

## Figures and Tables

**Table 1:** Hydrogen-bond geometry (Å, °)

*D*—H⋯*A*	*D*—H	H⋯*A*	*D*⋯*A*	*D*—H⋯*A*
N3—H3⋯N16^i^	0.86	2.05	2.907 (5)	172

Symmetry code: (i) 
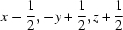
.

**Fig. 1.**
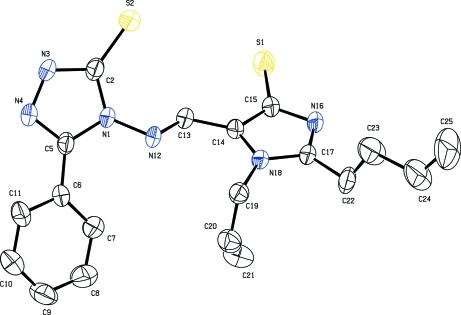
ORTEP diagram of the title molecule with the atom numbering scheme. H atoms were omitted. Displacement ellipsoid are drawn at 30% probability level.

**Fig. 2.**
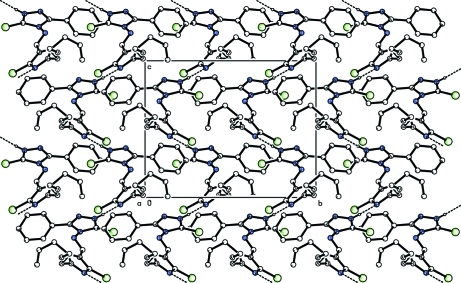
Packing diagram of the title compound viewed down the a axis. H atoms not involved in hydrogen bonds were omitted. Dashed lines indicate the intermolecular interactions between the molecules.

## supplementary crystallographic information

### Comment

Synthesis and structural investigation of Schiff base compounds have been given attention due to their interesting structural features and potential biological applications (Liang, 2003; Bacci *et al.*, 2005). The biological importance of imidazoles and triazoles has stimulated much work on these heterocycles. Triazole compounds and their derivatives have many applications in medicine and were reported to exhibit various pharmacological activities such as antimicrobial, analgesic, anti-inflammatory, anticancer and antioxidant properties (Amir *et al.*, 2008; Sztanke *et al.*, 2008; Padmavathi *et al.*, 2008; Thenmozhi *et al.*, 2010). The 1,2,4-triazole group interacts strongly with heme iron and aromatic substituents on the triazoles are very effective for interacting with the active site of aromatase. Furthermore, it was reported that pharmacological compounds having triazole moieties such as Vorozole, Anastrozole and Letrozole appear to be very effective aromatese inhibitors for preventing breast cancer (Ünver *et al.*, 2010). In view of these important applications of imidazolines, here we report the crystal structure of the title compound (Fig. 1).

The title compound contains imidazole and 1,2,4-triazole rings connected by an imine group. A phenyl ring is substituted at position 5 of the triazole ring and the dihedral angle between these rings is 24.1 (2)°. The imidazole and triazole groups are substituted on the imine group (N12—C13) in the E-configuration [N1—N12—C13—C14 = -174.4 (3)°]. The triazole ring is not co-planar with the imidazole ring and this may be due to the substitution of the phenyl ring on the triazole ring. The dihedral angles between the imidazole ring and the triazole and phenyl rings are 39.9 (2)° and 55.3 (2)°, respectively. The imidazole ring is substituted by bulky groups (3-butene, 2-propene) as well as an imine and a thiol group, which gives strain on the ring. The 3-butene and imine substituents show an extended zigzag confirmation with respect to the imidazole ring.

The packing diagram of the title compound viewed down the *a* axis is shown in Fig. 2. The crystal packing displays intermolecular N—H···N hydrogen bonds (Table 1), which join the molecules into chains in the [1 0 -1] direction.

### Experimental

The title compound was synthesized by refluxing 4-amino-5-phenyl-2,4-dihydro-3*H*-1,2,4-triazole-3-thione (0.01 mmol) and 2-(but-3-en-1-yl)-1-(prop-2-en-1-yl)-4-sulfanyl-1*H*-imidazole-5- carbaldehyde (0.01 mmol) in ethanol (50 ml) with a few drops of H_2_SO_4_ for 3 h on a water bath. The reaction progress was monitored by TLC. The resulting precipitate was filtered off, washed with cold ethanol, dried and purified to give the target product as a colorless solid in 74% yield. The resulting Schiff base compound was seperated out and crystallized in ethanol.

### Refinement

H atoms were positioned geometrically, taking H-bond formation potential into account where necessary, and refined using a riding model with C—H = 0.93 Å for aromatic H, 0.97 Å for methylene, for aromatic N—H = 0.86 Å and for S—H = 1.2 Å. The *U*_iso_ parameters for H atoms were constrained to be 1.5U_eq_ of the carrier atom for the thiol H atom and 1.2U_eq_ of the carrier atom for the remaining H atoms.

### Crystal data

**Table:**

C_19_H_20_N_6_S_2_	*F*(000) = 832
*M**_r_* = 396.53	*D*_x_ = 1.276 Mg m^−^^3^
Monoclinic, *C**c*	Mo *K*α radiation, λ = 0.71073 Å
*a* = 13.384 (3) Å	Cell parameters from 7754 reflections
*b* = 13.892 (3) Å	θ = 2.1–27.0°
*c* = 11.349 (2) Å	µ = 0.27 mm^−^^1^
β = 101.953 (3)°	*T* = 293 K
*V* = 2064.5 (7) Å^3^	Prism, pale yellow
*Z* = 4	0.28 × 0.25 × 0.23 mm

### Data collection

**Table:**

Bruker SMART APEX CCD area-detector diffractometer	3391 reflections with *I* > 2σ(*I*)
Radiation source: fine-focus sealed tube	*R*_int_ = 0.017
graphite	θ_max_ = 27.0°, θ_min_ = 2.1°
ω scans	*h* = −16→16
7754 measured reflections	*k* = −17→17
3788 independent reflections	*l* = −14→13

### Refinement

**Table:**

Refinement on *F*^2^	Primary atom site location: structure-invariant direct methods
Least-squares matrix: full	Secondary atom site location: difference Fourier map
*R*[*F*^2^ > 2σ(*F*^2^)] = 0.053	Hydrogen site location: inferred from neighbouring sites
*wR*(*F*^2^) = 0.139	H-atom parameters constrained
*S* = 1.07	*w* = 1/[σ^2^(*F*_o_^2^) + (0.0788*P*)^2^ + 0.8006*P*] where *P* = (*F*_o_^2^ + 2*F*_c_^2^)/3
3788 reflections	(Δ/σ)_max_ = 0.001
244 parameters	Δρ_max_ = 0.33 e Å^−^^3^
2 restraints	Δρ_min_ = −0.21 e Å^−^^3^

### Special details

**Table:**

Geometry. All e.s.d.'s (except the e.s.d. in the dihedral angle between two l.s. planes) are estimated using the full covariance matrix. The cell e.s.d.'s are taken into account individually in the estimation of e.s.d.'s in distances, angles and torsion angles; correlations between e.s.d.'s in cell parameters are only used when they are defined by crystal symmetry. An approximate (isotropic) treatment of cell e.s.d.'s is used for estimating e.s.d.'s involving l.s. planes.
Refinement. Refinement of *F*^2^ against ALL reflections. The weighted *R*-factor *wR* and goodness of fit *S* are based on *F*^2^, conventional *R*-factors *R* are based on *F*, with *F* set to zero for negative *F*^2^. The threshold expression of *F*^2^ > σ(*F*^2^) is used only for calculating *R*-factors(gt) *etc*. and is not relevant to the choice of reflections for refinement. *R*-factors based on *F*^2^ are statistically about twice as large as those based on *F*, and *R*- factors based on ALL data will be even larger.

### Fractional atomic coordinates and isotropic or equivalent isotropic displacement parameters (Å^2^)

**Table:**

	*x*	*y*	*z*	*U*_iso_*/*U*_eq_	
S1	0.62454 (8)	0.22573 (8)	−0.09079 (10)	0.0795 (3)	
H1	0.6713	0.1935	−0.1613	0.119*	
S2	0.50671 (9)	0.18382 (7)	0.24665 (11)	0.0776 (3)	
N1	0.4682 (2)	0.38001 (19)	0.2489 (3)	0.0520 (6)	
C2	0.4505 (3)	0.2855 (3)	0.2779 (3)	0.0607 (8)	
N3	0.3797 (2)	0.2949 (2)	0.3446 (3)	0.0677 (8)	
H3	0.3536	0.2465	0.3748	0.081*	
N4	0.3523 (3)	0.3875 (2)	0.3605 (3)	0.0695 (8)	
C5	0.4076 (3)	0.4390 (3)	0.3039 (3)	0.0565 (8)	
C6	0.3977 (3)	0.5438 (3)	0.2943 (3)	0.0607 (8)	
C7	0.4199 (3)	0.5949 (3)	0.1981 (4)	0.0718 (10)	
H7	0.4487	0.5641	0.1405	0.086*	
C8	0.3985 (4)	0.6924 (3)	0.1887 (5)	0.0930 (14)	
H8	0.4135	0.7268	0.1242	0.112*	
C9	0.3562 (4)	0.7392 (4)	0.2712 (6)	0.0979 (16)	
H9	0.3405	0.8043	0.2621	0.117*	
C10	0.3367 (5)	0.6889 (4)	0.3690 (6)	0.1026 (16)	
H10	0.3098	0.7205	0.4276	0.123*	
C11	0.3571 (4)	0.5923 (3)	0.3796 (5)	0.0864 (13)	
H11	0.3434	0.5588	0.4454	0.104*	
N12	0.5512 (2)	0.4157 (2)	0.2041 (2)	0.0534 (6)	
C13	0.5707 (2)	0.3656 (2)	0.1163 (3)	0.0510 (7)	
H13	0.5263	0.3157	0.0863	0.061*	
C14	0.6567 (2)	0.3821 (2)	0.0621 (3)	0.0498 (7)	
C15	0.6859 (3)	0.3256 (2)	−0.0233 (3)	0.0554 (8)	
N16	0.7739 (2)	0.3557 (2)	−0.0521 (3)	0.0586 (7)	
C17	0.8009 (3)	0.4324 (2)	0.0163 (3)	0.0576 (8)	
N18	0.7337 (2)	0.45065 (18)	0.0878 (2)	0.0529 (6)	
C19	0.7387 (3)	0.5317 (3)	0.1724 (3)	0.0615 (8)	
H19A	0.7062	0.5130	0.2378	0.074*	
H19B	0.8098	0.5459	0.2067	0.074*	
C20	0.6890 (4)	0.6192 (3)	0.1151 (5)	0.0787 (12)	
H20	0.6881	0.6715	0.1659	0.094*	
C21	0.6481 (5)	0.6321 (4)	0.0080 (6)	0.0983 (16)	
H21A	0.6465	0.5826	−0.0475	0.118*	
H21B	0.6191	0.6915	−0.0170	0.118*	
C22	0.8959 (4)	0.4875 (3)	0.0161 (5)	0.0925 (16)	
H22A	0.9024	0.4973	−0.0666	0.111*	
H22B	0.8902	0.5503	0.0514	0.111*	
C23	0.9883 (4)	0.4404 (4)	0.0821 (9)	0.152 (3)	
H23B	0.9879	0.3736	0.0571	0.182*	
H23A	0.9880	0.4415	0.1675	0.182*	
C24	1.0858 (6)	0.4886 (5)	0.0613 (15)	0.220 (6)	
H24	1.0844	0.5505	0.0298	0.264*	
C25	1.1675 (6)	0.4425 (8)	0.0880 (11)	0.199 (5)	
H25A	1.1677	0.3807	0.1195	0.239*	
H25B	1.2281	0.4700	0.0764	0.239*	

### Atomic displacement parameters (Å^2^)

**Table:**

	*U*^11^	*U*^22^	*U*^33^	*U*^12^	*U*^13^	*U*^23^
S1	0.0824 (7)	0.0741 (6)	0.0920 (7)	−0.0194 (5)	0.0409 (6)	−0.0316 (5)
S2	0.0906 (7)	0.0572 (5)	0.0970 (7)	−0.0004 (5)	0.0467 (6)	−0.0015 (5)
N1	0.0515 (14)	0.0552 (15)	0.0571 (15)	−0.0019 (11)	0.0294 (12)	0.0031 (12)
C2	0.0590 (19)	0.068 (2)	0.061 (2)	−0.0067 (16)	0.0263 (16)	0.0024 (15)
N3	0.0701 (18)	0.0648 (18)	0.081 (2)	−0.0029 (14)	0.0454 (16)	0.0120 (15)
N4	0.0696 (18)	0.0688 (18)	0.084 (2)	0.0036 (15)	0.0483 (17)	0.0082 (16)
C5	0.0526 (17)	0.070 (2)	0.0534 (18)	0.0031 (15)	0.0248 (14)	0.0057 (15)
C6	0.0495 (18)	0.0632 (19)	0.075 (2)	0.0059 (15)	0.0267 (16)	0.0011 (17)
C7	0.081 (2)	0.067 (2)	0.074 (2)	0.0027 (19)	0.031 (2)	0.0065 (18)
C8	0.111 (4)	0.076 (3)	0.095 (3)	0.005 (3)	0.027 (3)	0.019 (2)
C9	0.102 (4)	0.066 (3)	0.125 (4)	0.027 (2)	0.022 (3)	0.005 (3)
C10	0.112 (4)	0.082 (3)	0.127 (4)	0.024 (3)	0.057 (3)	−0.007 (3)
C11	0.092 (3)	0.087 (3)	0.095 (3)	0.023 (2)	0.053 (3)	0.002 (2)
N12	0.0522 (14)	0.0620 (15)	0.0540 (15)	−0.0017 (12)	0.0295 (12)	0.0045 (12)
C13	0.0480 (16)	0.0578 (18)	0.0504 (18)	−0.0032 (13)	0.0178 (14)	0.0021 (14)
C14	0.0515 (17)	0.0523 (16)	0.0499 (18)	0.0035 (13)	0.0208 (14)	0.0019 (13)
C15	0.0579 (18)	0.0545 (18)	0.060 (2)	0.0018 (14)	0.0274 (15)	−0.0023 (14)
N16	0.0628 (16)	0.0585 (16)	0.0652 (18)	−0.0012 (13)	0.0380 (14)	−0.0036 (13)
C17	0.063 (2)	0.0534 (17)	0.067 (2)	−0.0015 (15)	0.0368 (17)	0.0028 (15)
N18	0.0605 (15)	0.0483 (13)	0.0578 (15)	−0.0009 (12)	0.0303 (12)	0.0003 (12)
C19	0.066 (2)	0.0562 (18)	0.067 (2)	−0.0088 (15)	0.0261 (17)	−0.0108 (15)
C20	0.082 (3)	0.068 (2)	0.089 (3)	−0.002 (2)	0.024 (3)	−0.016 (2)
C21	0.108 (4)	0.073 (3)	0.106 (4)	0.014 (3)	0.006 (3)	−0.002 (3)
C22	0.095 (3)	0.082 (3)	0.125 (4)	−0.032 (3)	0.079 (3)	−0.029 (3)
C23	0.059 (3)	0.084 (3)	0.322 (11)	−0.019 (2)	0.063 (4)	−0.027 (5)
C24	0.093 (5)	0.090 (4)	0.514 (19)	−0.031 (4)	0.152 (8)	−0.058 (7)
C25	0.080 (4)	0.223 (10)	0.314 (14)	−0.025 (5)	0.087 (6)	−0.018 (10)

### Geometric parameters (Å, °)

**Table:**

S1—C15	1.711 (4)	C14—C15	1.365 (4)
S1—H1	1.2000	C14—N18	1.389 (4)
S2—C2	1.673 (4)	C15—N16	1.352 (4)
N1—C2	1.385 (5)	N16—C17	1.324 (5)
N1—C5	1.389 (4)	C17—N18	1.355 (4)
N1—N12	1.405 (4)	C17—C22	1.485 (5)
C2—N3	1.337 (5)	N18—C19	1.472 (4)
N3—N4	1.359 (4)	C19—C20	1.471 (6)
N3—H3	0.8600	C19—H19A	0.9700
N4—C5	1.292 (4)	C19—H19B	0.9700
C5—C6	1.463 (5)	C20—C21	1.239 (7)
C6—C11	1.380 (5)	C20—H20	0.9300
C6—C7	1.386 (5)	C21—H21A	0.9300
C7—C8	1.384 (6)	C21—H21B	0.9300
C7—H7	0.9300	C22—C23	1.461 (9)
C8—C9	1.356 (8)	C22—H22A	0.9700
C8—H8	0.9300	C22—H22B	0.9700
C9—C10	1.381 (8)	C23—C24	1.529 (8)
C9—H9	0.9300	C23—H23B	0.9700
C10—C11	1.370 (7)	C23—H23A	0.9700
C10—H10	0.9300	C24—C25	1.250 (13)
C11—H11	0.9300	C24—H24	0.9300
N12—C13	1.285 (4)	C25—H25A	0.9300
C13—C14	1.432 (4)	C25—H25B	0.9300
C13—H13	0.9300		
			
C15—S1—H1	109.5	N16—C15—S1	120.2 (2)
C2—N1—C5	107.9 (3)	C14—C15—S1	127.1 (3)
C2—N1—N12	127.4 (3)	C17—N16—C15	104.6 (3)
C5—N1—N12	122.3 (3)	N16—C17—N18	111.6 (3)
N3—C2—N1	102.6 (3)	N16—C17—C22	123.0 (3)
N3—C2—S2	127.2 (3)	N18—C17—C22	125.4 (3)
N1—C2—S2	130.1 (3)	C17—N18—C14	107.5 (3)
C2—N3—N4	114.2 (3)	C17—N18—C19	126.0 (3)
C2—N3—H3	122.9	C14—N18—C19	126.4 (3)
N4—N3—H3	122.9	C20—C19—N18	112.8 (3)
C5—N4—N3	105.1 (3)	C20—C19—H19A	109.0
N4—C5—N1	110.2 (3)	N18—C19—H19A	109.0
N4—C5—C6	122.4 (3)	C20—C19—H19B	109.0
N1—C5—C6	127.2 (3)	N18—C19—H19B	109.0
C11—C6—C7	118.8 (4)	H19A—C19—H19B	107.8
C11—C6—C5	118.4 (4)	C21—C20—C19	128.4 (4)
C7—C6—C5	122.6 (3)	C21—C20—H20	115.8
C8—C7—C6	119.2 (4)	C19—C20—H20	115.8
C8—C7—H7	120.4	C20—C21—H21A	120.0
C6—C7—H7	120.4	C20—C21—H21B	120.0
C9—C8—C7	121.6 (5)	H21A—C21—H21B	120.0
C9—C8—H8	119.2	C23—C22—C17	113.7 (4)
C7—C8—H8	119.2	C23—C22—H22A	108.8
C8—C9—C10	119.2 (4)	C17—C22—H22A	108.8
C8—C9—H9	120.4	C23—C22—H22B	108.8
C10—C9—H9	120.4	C17—C22—H22B	108.8
C11—C10—C9	119.9 (5)	H22A—C22—H22B	107.7
C11—C10—H10	120.1	C22—C23—C24	112.6 (7)
C9—C10—H10	120.1	C22—C23—H23B	109.1
C10—C11—C6	121.2 (5)	C24—C23—H23B	109.1
C10—C11—H11	119.4	C22—C23—H23A	109.1
C6—C11—H11	119.4	C24—C23—H23A	109.1
C13—N12—N1	113.1 (3)	H23B—C23—H23A	107.8
N12—C13—C14	123.8 (3)	C25—C24—C23	118.0 (9)
N12—C13—H13	118.1	C25—C24—H24	121.0
N14—C13—H13	118.1	C23—C24—H24	121.0
C15—C14—N18	103.6 (3)	C24—C25—H25A	120.0
C15—C14—C13	125.9 (3)	C24—C25—H25B	120.0
N18—C14—C13	130.3 (3)	H25A—C25—H25B	120.0
N16—C15—C14	112.7 (3)		
			
C5—N1—C2—N3	1.5 (4)	N1—N12—C13—C14	−174.4 (3)
N12—N1—C2—N3	164.0 (3)	N12—C13—C14—C15	173.5 (3)
C5—N1—C2—S2	−175.1 (3)	N12—C13—C14—N18	−0.4 (5)
N12—N1—C2—S2	−12.6 (6)	N18—C14—C15—N16	−0.8 (4)
N1—C2—N3—N4	−0.5 (4)	C13—C14—C15—N16	−176.0 (3)
S2—C2—N3—N4	176.2 (3)	N18—C14—C15—S1	178.4 (3)
C2—N3—N4—C5	−0.7 (4)	C13—C14—C15—S1	3.3 (5)
N3—N4—C5—N1	1.6 (4)	C14—C15—N16—C17	0.1 (4)
N3—N4—C5—C6	177.0 (3)	S1—C15—N16—C17	−179.2 (3)
C2—N1—C5—N4	−2.0 (4)	C15—N16—C17—N18	0.7 (4)
N12—N1—C5—N4	−165.6 (3)	C15—N16—C17—C22	178.4 (4)
C2—N1—C5—C6	−177.2 (4)	N16—C17—N18—C14	−1.3 (4)
N12—N1—C5—C6	19.2 (5)	C22—C17—N18—C14	−178.8 (4)
N4—C5—C6—C11	23.2 (6)	N16—C17—N18—C19	−178.2 (3)
N1—C5—C6—C11	−162.2 (4)	C22—C17—N18—C19	4.2 (6)
N4—C5—C6—C7	−151.5 (4)	C15—C14—N18—C17	1.2 (3)
N1—C5—C6—C7	23.0 (6)	C13—C14—N18—C17	176.1 (3)
C11—C6—C7—C8	−1.5 (6)	C15—C14—N18—C19	178.1 (3)
C5—C6—C7—C8	173.2 (4)	C13—C14—N18—C19	−7.0 (5)
C6—C7—C8—C9	−0.1 (8)	C17—N18—C19—C20	86.8 (4)
C7—C8—C9—C10	1.9 (9)	C14—N18—C19—C20	−89.6 (4)
C8—C9—C10—C11	−2.1 (9)	N18—C19—C20—C21	−2.4 (7)
C9—C10—C11—C6	0.4 (9)	N16—C17—C22—C23	−76.5 (6)
C7—C6—C11—C10	1.4 (7)	N18—C17—C22—C23	100.8 (5)
C5—C6—C11—C10	−173.5 (5)	C17—C22—C23—C24	169.4 (7)
C2—N1—N12—C13	47.3 (4)	C22—C23—C24—C25	−162.9 (12)
C5—N1—N12—C13	−152.4 (3)		

### Hydrogen-bond geometry (Å, °)

**Table:**

*D*—H···*A*	*D*—H	H···*A*	*D*···*A*	*D*—H···*A*
N3—H3···N16^i^	0.86	2.05	2.907 (5)	172

Symmetry codes: (i) *x*−1/2, −*y*+1/2, *z*+1/2.
